# Allelic Differences within and among Sister Spores of the Arbuscular Mycorrhizal Fungus *Glomus etunicatum* Suggest Segregation at Sporulation

**DOI:** 10.1371/journal.pone.0083301

**Published:** 2013-12-26

**Authors:** Eva Boon, Erin Zimmerman, Marc St-Arnaud, Mohamed Hijri

**Affiliations:** Institut de recherche en biologie végétale, Département de sciences biologiques, Université de Montréal, Montreal, Quebec, Canada; University of Nebraska, United States of America

## Abstract

Arbuscular mycorrhizal fungi (AMF) are root-inhabiting fungi that form mutualistic symbioses with their host plants. AMF are made up of coenocytic networks of hyphae through which nuclei and organelles can freely migrate. In this study, we investigated the possibility of a genetic bottleneck and segregation of allelic variation at sporulation for a low-copy Polymerase1-like gene, *PLS*. Specifically, our objectives were (1) to estimate what allelic diversity is passed on to a single spore (2) to determine whether this diversity is less than the total amount of variation found in all spores (3) to investigate whether there is any differential segregation of allelic variation. We inoculated three tomato plants with a single spore of *Glomus etunicatum* each and after six months sampled between two and three daughter spores per tomato plant. Pyrosequencing *PLS* amplicons in eight spores revealed high levels of allelic diversity; between 43 and 152 alleles per spore. We corroborated the spore pyrosequencing results with Sanger- and pyrosequenced allele distributions from the original parent isolate. Both sequencing methods retrieved the most abundant alleles from the offspring spore allele distributions. Our results indicate that individual spores contain only a subset of the total allelic variation from the pooled spores and parent isolate. Patterns of allele diversity between spores suggest the possibility for segregation of *PLS* alleles among spores. We conclude that a genetic bottleneck could potentially occur during sporulation in AMF, with resulting differences in genetic variation among sister spores. We suggest that the effects of this bottleneck may be countered by anastomosis (hyphal fusion) between related hyphae.

## Introduction

Arbuscular mycorrhizal fungi (AMF) are root-inhabiting fungi that form mutualistic symbioses with plants and are grouped in the phylum Glomeromycota [1,2]. They improve nutrient uptake in their host plants and buffer the plant against both abiotic and biotic stresses [3-5]. As a consequence of these characteristics, AMF significantly increase plant growth rates, with benefits varying depending on the composition of both the fungal and plant communities [6]. AMF are of great potential interest to agriculture, and in recent years much progress has been made in understanding the peculiar genetics and reproductive biology of these organisms. 

Arbuscular mycorrhizal fungi are made up of vast, branching networks of hyphae. These hyphae are coenocytic, or lacking discrete cellular divisions. Hyphal walls form long, tube-like structures through which cytoplasm and organelles can freely migrate. At sporulation, spores are formed as outgrowths from the parent hyphae, and large numbers of nuclei migrate directly into the spores [7,8]. 

Mature AMF spores therefore contain hundreds of nuclei obtained directly from the parent mycelium, making these fungi, to our knowledge, the only organisms that do not pass through a uninucleate stage in any part of their life cycle [7-10]. This is significant, because the uninucleate stage has been hypothesized to act as a genetic bottleneck that prevents genetic conflict by ensuring that each daughter cell possesses only one copy of the original genome [11]. Furthermore, if one considers that a single nucleus versus a multinuclear state could be evolutionarily analogous to a haploid versus a polyploid genome, a uninuclear state might allow for more efficient purging of deleterious alleles [12] .

The prevention of genetic conflict is at the heart of many theories of individuality [11,13,14]. Nevertheless, strict soma-germline division is applicable only to metazoans. Many other multicellular organisms, such as plants, fungi, and algae, do not have such a clear-cut soma-germline division and consequent bottleneck in their life history. Accordingly, the extent of intra-individual genetic heterogeneity among multicellular organisms may be vastly underestimated [15-17]. 

In an organism that lacks a uninucleate stage, genome polymorphisms due to somatic mutations are allowed to pass on to the next generation, resulting in individuals that can theoretically contain any number of differentiated genomes [18]. Indeed, numerous studies confirm that alleles can vary within and between AMF isolates and sister spores, with evidence coming from rRNA genes (rDNA) [19-24], protein-coding genes [20,22,25-28], and even from the transcriptome [22,29]. 

What evidence is there for the heterokaryotic state in AMF? A 2001 study by Kuhn et al. [20] used double-target fluorescent DNA-DNA *in situ* hybridization to visualize two alleles of the ITS2 region, T2 and T4. These two alleles were found to occur in varying frequencies in different nuclei, indicating non-identical genomes. A similar experiment was performed for the protein-coding gene BiP [30]. Furthermore, a large discrepancy between copy number per nucleus and allele number per isolate indicated that, for Large Subunit (LSU) rDNA, allele variants were partitioned between and not within nuclei in the same isolate [22]. Finally, Ehinger et al. [31,32] found genotypic and phenotypic variation after clonal growth of spores over three successive generations, likely with corresponding variation in fitness [32]. 

Much of the heterokaryosis discussion has revolved around the marker *PLS* (*Polymerase 1*-like sequence). Pawlowska and Taylor proposed in 2004 [33] that segregation patterns of 13 *PLS* alleles among sister spores support the theory of homokaryosis, i.e. that nuclei within the *G. etunicatum* cytoplasm are polyploid and genetically homogeneous. This publication initiated a discussion on the correct interpretation of the data, in which Bever et al. [34] contested that low rates of hyphal fusion could maintain a heterokaryotic state in AMF. In their reply, Pawlowska and Taylor [35] 'explicitly excluded the possibility that vegetative hyphal fusions among genetically differentiated individuals could contribute to the creation and maintenance of multigenomic individuals of AM fungi'. However, subsequent studies have pointed to the possible role of anastomosis in the exchange of genetic variation between AMF isolates [32,36-39].

Two recent studies that support the heterokaryosis theory studied the *PLS* marker in *G. etunicatum*. First, Hijri and Sanders [40] showed that *G. etunicatum* is haploid and *PLS* is present in two copies in the genome. They proposed that *PLS* variants were likely partitioned among different nuclei. Second, variation seen at the genomic level was found to persist in the cDNA, which indicates that high allele numbers are not simply attributable to a high number of *PLS* pseudogenes in the AMF genome [22]. 

**Figure 1:**
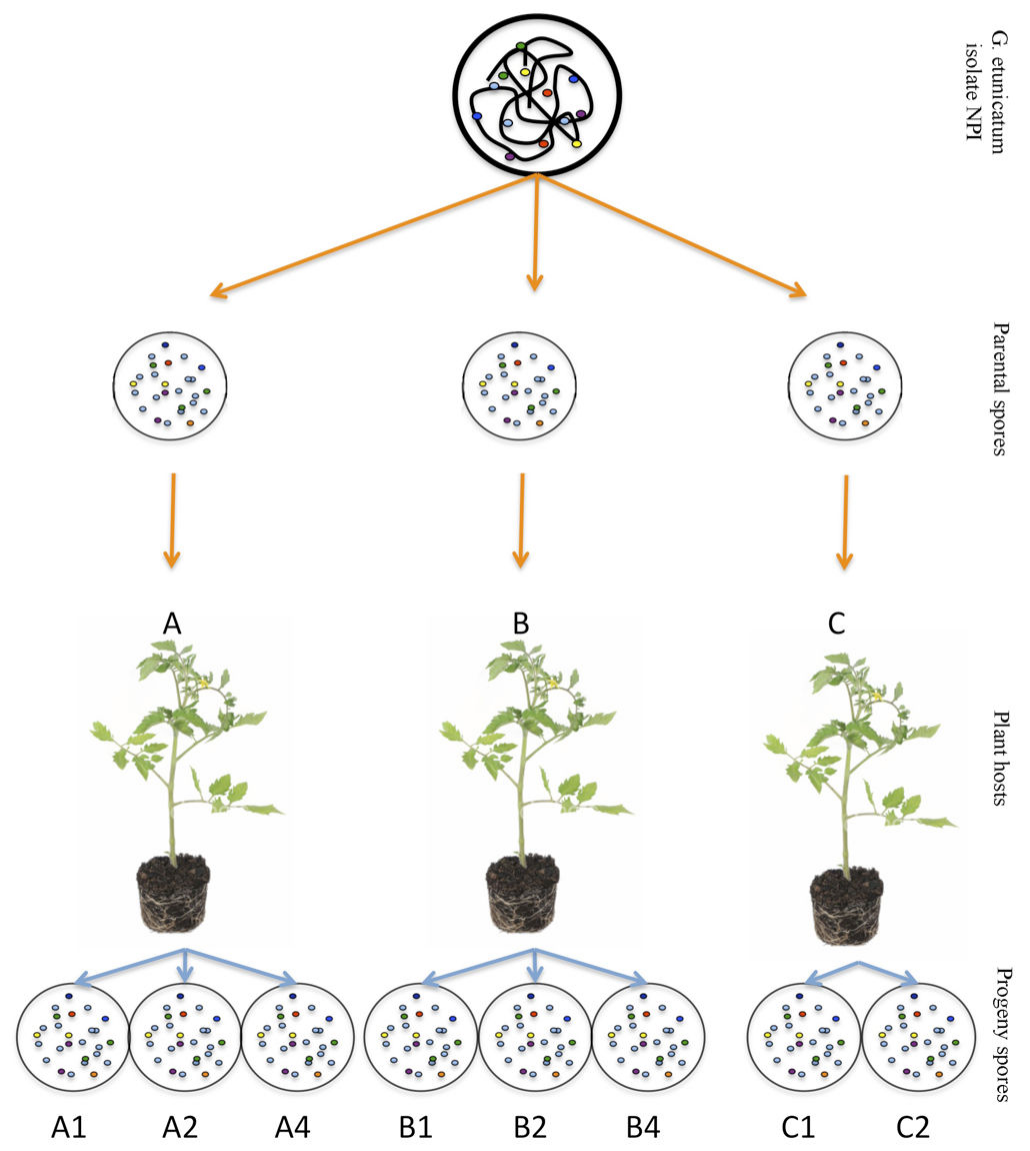
Diagram of the experimental setup. Three parent spores were taken from a single isolate of *G. etunicatum* and grown in pot culture to produce eight progeny spores for pyrosequencing.

In the present study, we explore the extent of within-isolate genetic polymorphism for *PLS* in *Glomus etunicatum* and investigate whether a bottleneck of allelic variation and segregation occur at sporulation. This was accomplished by inoculating tomato plants with individual spores (Figure 1). Eight daughter spores were isolated from three such cultures; from each, we amplified a fragment from *PLS*. As mentioned above, several markers are found to be polymorphic among nuclei in AMF. We chose the *PLS* marker because it is present in low copy numbers in the *G. etunicatum* genome [40], and we would therefore be less likely to confound intra-genome with inter-genome variation. Amplicon products from the selected daughter spores were sequenced using Roche FLX Titanium sequencing and verified with Sanger sequencing. In this way, allelic variation within each spore could be compared to that of spores from the same plant, or to all spores together. 

Our specific objectives were (1) to estimate what proportion of allelic diversity is passed on to individual spores (2), to determine whether this proportion is less than the total amount of variation found in all spores combined, and (3) to investigate whether segregation occurs at sporulation. 

## Results

### Pyrosequencing

All analyses were performed with Mothur v.1.22.0 [41], unless otherwise noted. Stringent checks were performed on the raw pyrosequencing data (131,797 reads) to remove low-quality reads and minimize sequencing errors that may have been introduced during the pyrosequencing process [42,43]. Quality control criteria and procedure are described in the Materials and Methods. After quality control, 14,320 reads were retained for eight spores, which represents approximately 11% of the original data (Table 1 and S1). The retained alignment covers 200 bp of the *PLS* gene, including the end of the first exon, the entire first intron, and the beginning of the second exon, according to the structure of the gene as previously described in Boon et al. [22].

**Table 1:** Diversity estimates for the 2^nd^ exon sequence of *PLS*.

		# reads											
		before		after	% reads retained					Size bottleneck^1^ (%)			
		quality control				# alleles	Chao1	Chao1 lci	Chao1 hci		95 % lci	95 % hci	
All		131797	14320		11	356	813	664	1036	56	46	66	
Plants	A	65274	5767		9	209	538	398	780	61	48	73	
	B	49704	5928		12	196	361	292	479	46	33	59	
	C	16819	2625		16	120	231	180	328	48	33	63	
Spores	A1	11242	1052		9	68	154	105	267	56	35	74	
	A2	43001	3811		34	152	314	240	452	52	37	66	
	A4	11031	904		2	87	209	147	333	58	41	74	
	B1	22711	3585		32	135	218	179	293	38	25	54	
	B2	5051	1536		7	82	170	124	267	52	34	69	
	B4	21942	807		16	79	229	150	398	66	47	80	
	C1	11920	2032		9	98	191	145	282	49	32	65	
	C2	4899	593		5	43	118	72	237	64	41	82	

^1^ Reduction of the number of alleles in comparison with the Chao1 index for the group, its lower confidence interval (lci) and higher confidence interval (hci).

The remaining reads represented 356 unique sequences (henceforth referred to as ‘alleles’). A clear asymptote in the rarefaction analysis is an indication that sampling was representative of the actual diversity. We performed rarefaction analyses for all spores together, ordered by plant, or individually (Figure S1). Given that the intron represented most of the allelic diversity (Figure S2) and the rarefaction analysis showed we had not exhaustively sampled all allelic diversity over the complete alignment, we excluded the intron. Even with this shortened alignment, none of the rarefaction curves reached the lower confidence interval of the Chao1 diversity index, which is a measure of the minimum richness in a sample [44] (Table 1, Figure S1). We concluded we had not exhaustively sampled all allelic diversity in our dataset and performed the remaining analyses on the exonic sequences.

Similarity in allelic diversity between spores, as measured by the shared Chao1 richness estimate for an allele (indicated with the abbreviation Sharedchao), the Jaccard similarity coefficient (jclass) based on the observed richness, and the Yue & Clayton (thetaYC) measure of dissimilarity between the structures of two communities, was tested with the UniFrac, AMOVA and HOMOVA tests. Weighted UniFrac scores were significantly different between all spore pairs, while most comparisons with spore A1 were significant between spores applying the AMOVA and HOMOVA tests (Table 2). Allelic diversity among plants was significantly different when applying the pairwise weighted UniFrac test (Table 3). Finally, applying a Kolmogorov-Smirnov test, only the comparison of allele distributions between spores A1-B4 was significantly different, and none of the allele distributions between plants were significantly different (Tables 2 and 3).

**Table 2:** Tests for similarity of *PLS* allele diversity between spores.

Comparison		p-values^1^			Kolmogorov-Smirnov^5^
		UNIFRAC^2^	AMOVA^3^	HOMOVA^4^	α =0.01
A1-A2-A4-B1-B2-B4-C1-C2		**<0.0010**	**0.002**	**0.002**	n.a.
A1-A2		**<0.0010**	0.998	0.003	0
A1-A4		**<0.0010**	1	**0.001**	0
A1-B1		**<0.0010**	1	**0.001**	0
A1-B2		**<0.0010**	1	**0.001**	0
A1-B4		**<0.0010**	1	**0.001**	7
A1-C1		**<0.0010**	1	**0.001**	0
A1-C2		**<0.0010**	0.026	**0.001**	0
A2-A4		**<0.0010**	0.371	0.957	0
A2-B1		**<0.0010**	0.042	0.092	0
A2-B2		**<0.0010**	0.249	0.387	0
A2-B4		**<0.0010**	0.204	0.549	0
A2-C1		**<0.0010**	0.243	0.494	0
A2-C2		**<0.0010**	0.01	0.007	0
A4-B1		**<0.0010**	0.994	0.007	0
A4-B2		**<0.0010**	0.957	0.103	0
A4-B4		**<0.0010**	0.876	0.386	0
A4-C1		**<0.0010**	0.888	0.398	0
A4-C2		**<0.0010**	0.006	0.115	0
B1-B2		**<0.0010**	0.972	0.543	0
B1-B4		**<0.0010**	0.976	0.033	0
B1-C1		**<0.0010**	0.798	0.013	0
B1-C2		**<0.0010**	0.011	0.028	0
B2-B4		**<0.0010**	0.94	0.123	0
B2-C1		**<0.0010**	0.852	0.042	0
B2-C2		**<0.0010**	0.006	0.122	0
B4-C1		**<0.0010**	0.825	0.961	0
B4-C2		**<0.0010**	0.041	0.121	0
C1-C2		**<0.0010**	0.024	0.082	0

Significant p-values are shown in bold.

^1^ α after Bonferroni correction for multiple tests = 0.002

^2^ UNIFRAC test describes whether the communities have the same structure by chance [59,67].

^3^ Analysis of Molecular Variance (AMOVA) [60-62] determines whether the genetic diversity within each community is significantly different from the average genetic diversity of both communities pooled together [68].

^4^ Homogeneity of Molecular Variance (HOMOVA) [63] tests whether the genetic diversity between spores is homogeneous.

^5^ Two-sample Komogorov-Smirnov test with the null hypothesis that the allele distributions of the spores under comparison are the same. Specified in the table is the number of alleles for which the null hypothesis is rejected at α=0.01.

**Table 3:** Tests for similarity of *PLS* allele distribution and richness between plants.

	p- value^1^			
	A-B-C	A-B	A-C	B-C
UNIFRAC^2^				
Sharedchao^3^	1	**<0.001**	**<0.001**	**<0.001**
Jclass^4^	1	**<0.001**	**<0.001**	**<0.001**
Thetayc^5^	0.46	**<0.002**	**<0.001**	**<0.001**
Kolmogorov-Smirnov^6^	n.a.	n.s.	n.s.	n.s.

n.a. means not applicable; n.s. means not significant.

^1^ Significant p-values are shown in bold: experimental-wise error rate = 0.05.

^2^ UNIFRAC [59,67].

^3^ Shared Chao-1 richness estimate for an OTU definition [44].

^4^ Jaccard index describing the dissimilarity between two communities.

^5^ Yue & Clayton measure of dissimilarity between the structures of two communities.

^6^ Two-sample Komogorov-Smirnov test with the null hypothesis that the allele distributions of the spores under comparison are the same.

**Figure 2:**
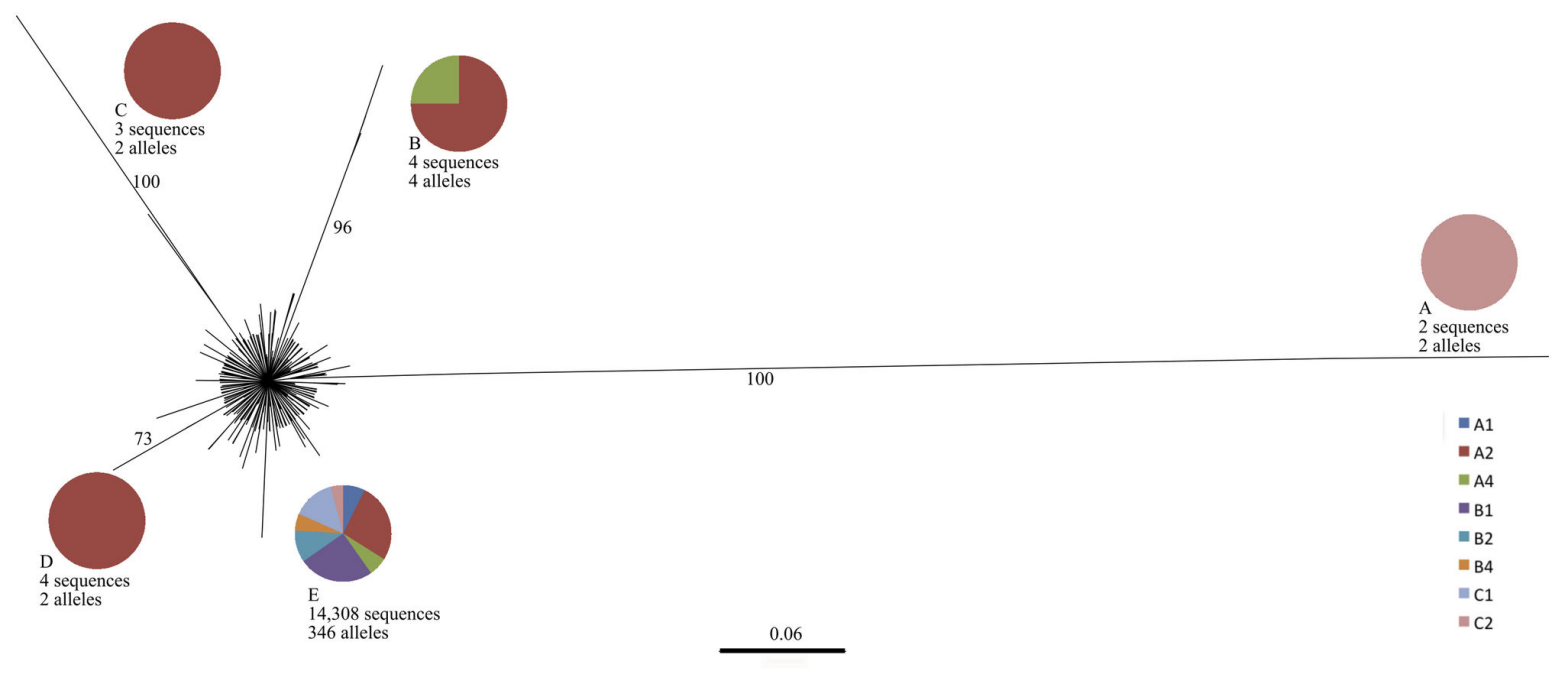
Phylogenetic analysis of genetic divergence between alleles (n=356, f=14,321, 92 nucleotide sites were used). Evolutionary relationships between alleles were inferred by maximum likelihood based on the Hasegawa-Kishino-Yano model of DNA evolution [46] with a discrete gamma distribution (5 categories (+G, parameter = 11.0677)). Statistical significance was tested with bootstrap replicates (n=1000); only values higher than 70 are depicted. Scale bar indicates number of substitutions per site. Pie charts indicate relative allele provenance for each allele group, as defined by bootstrap values >70.

 To further investigate the genealogical relations between alleles, we estimated the best model of codon evolution in jModelTest v.0.1.1 [45] and constructed a Maximum Likelihood tree in PhyML v3.0, under the Hasegawa-Kishino-Yano model of DNA evolution [46] with a discrete gamma distribution (5 categories (+G, parameter = 11.0677)), performing 1000 bootstrap replicates to test the significance of branch lengths. Most alleles were not significantly divergent (Figure 2). The alleles fell into five different groups named A-E with significant bootstrap support greater than 70%, which are depicted in Figure 2 with pie charts indicating spore provenance. Out of 356 alleles, 346 belonged to group E, which represents 14,308 sequences. Allele distributions of the most abundant *PLS* alleles recovered from pyrosequencing runs on spores and the parent isolate are depicted in Figure S3. The ten alleles that did not fall into group E represented only thirteen sequences, from spores A2, A4, and C2.

Finally, using the software DNAsp v.5 [47], we compared the translated amino acid sequences and found 921 stop codons and 19 frameshift mutations in the entire dataset. Of the 13 sequences that did not belong to group E, ten were found to contain stop codons. All results are summarized in Table 4.

**Table 4:** Changes on the amino acid level for *PLS*.

Group	Total alleles (# sequences)	Stop codon alleles (# sequences)
All	356 (14321)	54 (921)
A	2 (2)	2 (2)
B	4 (4)	3 (5)
C	2 (3)	2 (3)
D	2 (4)	0 (0)
E	346 (14308)	47 (911)

Group letters correspond to significantly differentiated groups from Figure 2. Numbers between brackets indicate total number of sequences retrieved per allele. Sequences with frameshift mutations are not depicted separately since they invariably corresponded to sequences that also contained stop codons.

### Sanger sequencing

To validate the allelic diversity observed in our pyrosequencing dataset, PCR and cloning was performed directly on total DNA extracted from thousands of spores of the *G. etunicatum* parent isolate, from which spores the experiment had been set up (isolate NPI). In total, 306 clones were sequenced, of which 167 covered the area that had also been retrieved by pyrosequencing. The remainder produced partial sequences or fragments of the plasmid. These 167 sequences resolved in four alleles, which grouped with the four most abundant alleles in the pyrosequencing dataset. When the first region of the second exon was evaluated at the same clustering level that had been employed for the pyrosequencing data (1%), the Sanger sequences still yielded four alleles that were all present at high frequencies in the pyrosequencing data. This confirms that at least the most frequent alleles observed in the pyrosequencing dataset are not due to whole genome amplification artefacts or pyrosequencing errors. Since the sampling intensity for pyrosequencing and Sanger sequencing is not comparable, we did not expect to recover all alleles found in the pyrosequencing dataset. Thus, even though finding the most abundant alleles in the Sanger sequencing is encouraging, we cannot be conclusive about whether the pyrosequencing alleles that were less abundant are artefactual or not.

We extracted 182 *PLS1* sequences from the 306 clones for a comprehensive analysis of a larger part of the *PLS* marker. If we assumed that every new single nucleotide polymorphism (SNP) represented a different allele, we found 113 sequences containing 66 variable sites. Using more conservative criteria, in which all SNPs occurring in only one sequence were dismissed as artefactual, we found 103 distinct sequences containing 38 variable sites. For both estimates, the three most abundant variants make up 11.5% of all sequences, while singletons make up 69.9% and 64.1% of the distinct sequences in conventional (1 SNP = 1 allele) and conservative (i.e. alleles are only counted if SNP occurs more than once) estimates, respectively. Twelve of the sequences contain an identical 5 bp insertion in the non-coding region following the third exon. Results are summarized in Table S2.

**Figure 3:**
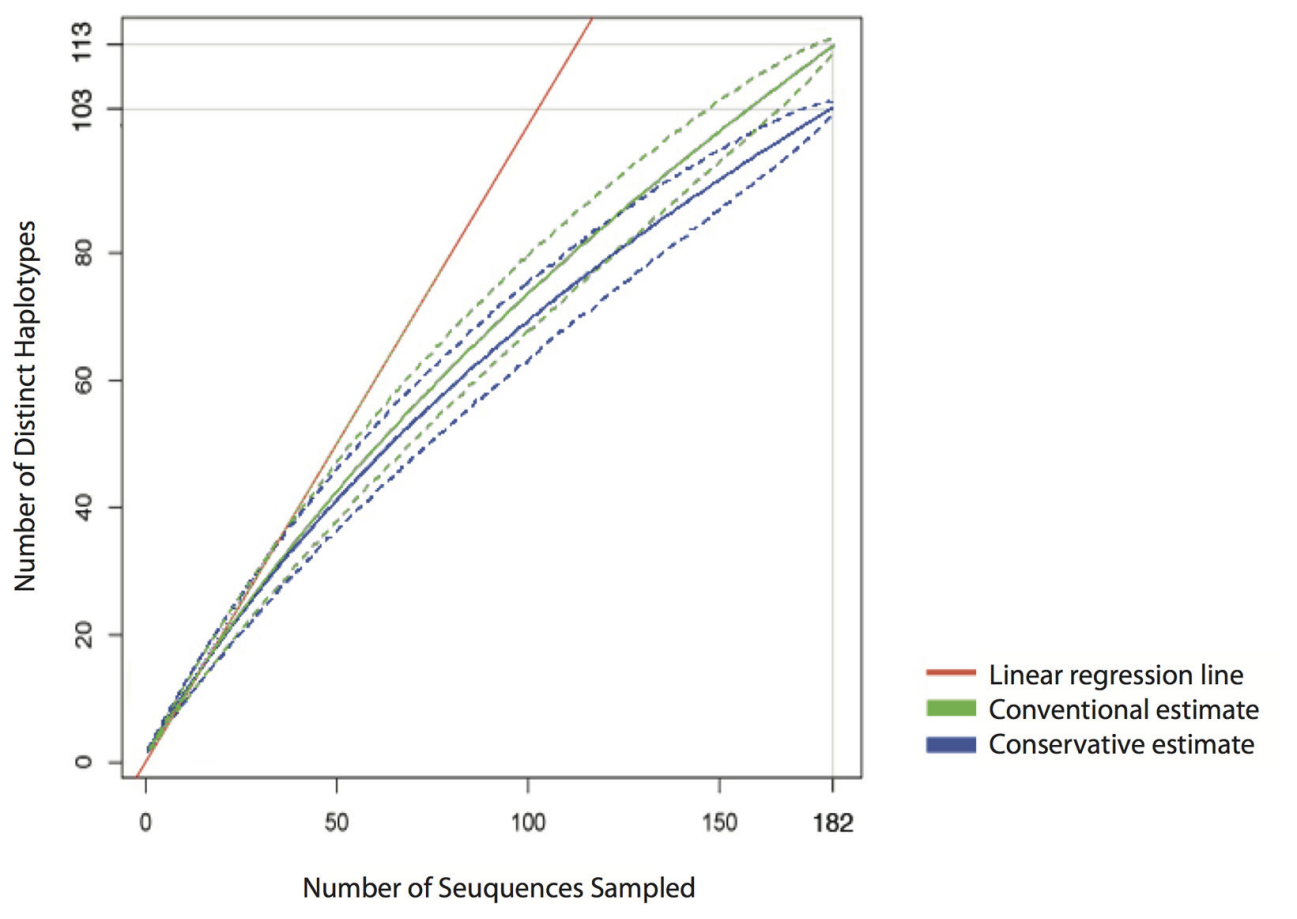
Monte Carlo simulations showing distinct variant numbers of *PLS* sequences, for both conventional (every new SNP leads to a different allele) and conservative (alleles are only counted if SNPs occurs more than once) estimates (1000 replicates). Dashed lines show 95% confidence intervals of the mean of the simulated values.

 Under the conservative estimate, only two sequences contained stop codons in their amino acid sequences, both caused by a single frameshift mutation; all other sequences were putatively functional. Of the 180 translated sequences, 49 were unique based on 19 variable sites. We resampled the allele variation with Monte Carlo simulations for the full-length *PLS* sequences obtained by Sanger sequencing. Since the sampling curve did not reach a plateau, we concluded that the allelic diversity had not been sampled exhaustively (Figure 3). 

## Discussion

Our first objective was to estimate the proportion of genetic diversity that is passed on in a single *G. etunicatum* spore for the marker *PLS*. Using both pyrosequencing and cloning of PCR products combined with Sanger sequencing, we found an unexpectedly high allelic diversity within individual spores. Both sequencing approaches revealed high numbers of single nucleotide polymorphisms (SNPs). 

When we compared total allele diversity from all spores pooled together to that found per individual spore, as estimated with the Chao1 statistic, we found a loss of allelic diversity as high as 41-82% for C2, the spore with the lowest allele diversity, and 37-66% for A2, the spore with the highest allele diversity (Table 1). 

We further attempted to determine whether the proportion of allelic diversity that ends up in a spore after sporulation is less than the total amount of variation found in all spores combined. In other words, can a single spore contain all variation found in all spores pooled together? Based on the smallest Chao1 estimates of between 72 and 237 alleles for spore C2, an individual spore would not be expected to contain all the allelic diversity of the parent isolate in our experiment. Even the allelic diversity estimate for spore A2, which with between 240 and 452 alleles yielded the most diversity, is smaller than the estimated minimum number of alleles from all spores pooled together (664 to 1036 alleles). Therefore, in our data, an individual spore always contains fewer alleles than the overall allelic diversity observed in eight spores pooled together.

We then proceeded to compare the pyrosequenced allele diversity found in the parent isolate, which produced the spores used to inoculate tomato plants, to the allele diversity found in the spores. Again, rarefaction analyses and the Chao1 index indicated that diversity for the *PLS* locus was not sampled exhaustively (Figure S4). Out of 96 sequences, eight alleles were retrieved. Out of these alleles, two remained after preclustering at the same 1% level as the alleles from the offspring spores. These remaining two alleles belonged to the most abundant alleles of those from the offspring spores. Sequencing coverage for the parent isolate was much lower compared to coverage of the spores (2,422 sequences were retrieved from the parent isolate vs. 131,797 sequences from the pooled offspring spores). Thus, neither parent nor offspring spores were exhaustively sampled and possible sampling effects made it uninformative to test for significant differences between allele distributions from spores and parent.

Finally, we investigated whether differential segregation of genetic variation occurs. We tested this by comparing allele distributions between spores, using a range of statistical methods to test the significance of the differences between distributions (Tables 2 and 3). If allele distributions are significantly different from each other, and no subsequent anastomosis takes place, differential segregation has occurred between allele populations in the parent and progeny. Although the significance of the tests varied with the estimator and the spore under consideration, the most abundant alleles were shared by all spores (Figure 2) and were also found in the reads obtained via Sanger sequencing. Unfortunately, we cannot draw any conclusions about differential segregation of more rare alleles, since our allelic diversity was not exhaustively sampled. However, the confidence intervals of the Chao1 estimator do allow us to detect a significant difference between the total amount of allele diversity at the spore level and the total amount of allele diversity observed in all spores together. In summary, even though we cannot offer conclusive evidence of segregation of *PLS* alleles in *G. etunicatum*, we do not expect that more exhaustive sampling of parent and spore allele diversity will compromise our suggestions, based on the present dataset.

The high polymorphism we found in *PLS* alleles for Sanger and pyrosequencing is not due to artefacts. In a previous study, we found similar allelic diversity patterns when comparing *PLS* alleles obtained from amplicon pyrosequencing with results obtained from PCR cloning and Sanger sequencing [22]. Moreover, the four most abundant alleles from our pyrosequencing results were also found in 167 *PLS* sequences obtained from Sanger sequencing. An additional 58 *PLS* sequences retrieved from GenBank, from a previous study [35], also matched the alleles found in this study. However, it remains important to review the role of error and bias in our analysis. 

Error control in next generation sequencing has become a widely discussed pitfall, especially for pyrosequencing. Huse et al. [42] were the first to address the question of pyrosequencing error, and these authors later proposed a preclustering methodology to prevent inflated diversity counts in pyrosequencing datasets [48]. Other authors tested for error rate on the more recent Titanium GS FLX platform that we used for our samples, and reported a mean error rate of 1.07 % [49]. With error rates this high, the utmost care is warranted in the treatment of pyrosequencing samples. 

We are not alone in advocating this conservative approach. The creators of Mothur, the pipeline we used to analyse our pyrosequencing samples, discard 99% of their sequences in an online tutorial (http://www.mothur.org/wiki/Schloss_SOP). Note that this number is so high because only unique sequences are counted. In a recent paper, simulations were used to explore the effect of various quality control measures on the error rate in pyrosequencing data. It was found that eliminating 63.5% of the raw sequence reads reduced the error rate from 0.0061 to 0.0007 [43]. The various effects of removing chimeric sequences (~40%, [50]), quality filtering (~30%, [51]) and preclustering [48] easily add up, and the removal of high percentages of raw sequence data up to 98% are routinely reported [52]. 

Thus, it is important to verify potential bias. In our experiment, all offspring spores were sequenced in the same run, avoiding run-quality bias. We also calculated bias in quality control, removal of chimeric sequences and preclustering (Table S1). In total, between 97% and 99%% of raw sequence data was removed for each spore. These percentages were not significantly different between spores (average proportion of sequences retained ± st.dev.; 0.02 ± 0.01). It is possible that the large numbers of reads we removed may have led to our failure to exhaustively sample all allele variation at the *PLS* locus on the spore and plant levels. Future studies on the subject of allele diversity in AMF should take advantage of improvements in sequencing technology to sample AMF diversity with a greater depth and statistical power than we were able to achieve here.

We would also like to briefly discuss the applicability of diversity estimators that are originally derived from ecological theory to our data. The depth of our sampling was estimated using the Chao1 index, which is based on singletons and doubletons in the data [44]. The dissimilarity between communities was described with the Jaccard index, which describes the dissimiliarity between communities based on (in this case) allele counts [44]. Community structure was described with the Yue & Clayton measure of dissimilarity, which is based on relative abundances [53]. None of these beta-diversity estimators are based on variables that are exclusive to an ecological context. Moreover, these estimators are often used in microbial community studies where the community is not always clearly defined. Therefore, we are confident that the beta-diversity estimators here are used in an appropriate way. 

In spite of the extraordinary amount of allelic diversity that was found in the spores, we propose that the large majority of *PLS* alleles that we investigated are under functional constraints, for the following reasons. First, the exon of the *PLS* gene shows considerably less nucleotide diversity than the intron (Figure S2). Second, only 14-15% of alleles were found to contain stop codons (Table 4). Finally, most alleles were found not to be significantly different from each other, based on a maximum likelihood analysis (Figure 2). The alleles that did vary significantly were present at very low frequencies and in most cases contained stop codons, which means these alleles are not transcribed. The large amount of allelic diversity in *G. etunicatum*, which translates into little amino acid diversity, confirms previous reports [22]. 

If the number of *PLS* alleles that are present in the parent mycelium is larger than the average number of *PLS* alleles in a daughter spore, it is unlikely that the daughter spore will receive the full genetic complement of the parent, a process which we refer to as segregation. This study provides indications that a single spore is not sufficient to transfer all genetic variation that was present in the parent cytoplasm to the next generation, which means that *G. etunicatum* is potentially exposed to a loss of genetic variation at sporulation, as was originally proposed by Pawlowska et al. [33]. Without replenishment of genetic variation, this segregation leads to a loss of nuclear variants with each new generation and a loss of heterokaryosis over time. This process was modelled by Bever and Wang [9,34], who found that the only means of countering this loss is through the action of anastomosis, in which hyphal strands, either within or among mycelia, fuse and nuclei are exchanged. If we place a bottleneck of the magnitude we observed in this study (40%) in the context of the Bever et al. model [9,34], we find that without anastomosis, all allelic diversity is expected to be lost in ten generations, whereas with 2-25% anastomosis, most allelic diversity is conserved.

Segregation of genetically differentiated nuclei could lead to different genomic contents between subcultures from the same isolate, and recent research has demonstrated that genetic drift may occur. *Glomus irregulare* isolate DAOM 197198 has been maintained in several different *in vitro* culture collections since 1992. Cardenas-Flores et al. [54] found that subcultures from the same isolate which had been maintained in separate laboratories for 69 generations anastomosed at a much lower rate than spores taken from within the same isolate and the same subculture. This could indicate that subcultures no longer share the same genome content, since decreased rates of anastomosis are observed between isolates that are genetically more different [8,38,55,56]. A recent report demonstrated that a significant genetic variability (difference in allelic frequencies of the Bg112 locus) occurred from a clonal growth of single spores of *G. irregulare* over three generations [31]. Differential segregation of nuclei at sporulation could lead to rapid genetic differentiation between subcultures and could effectively extinguish intra-isolate genetic variation within a few generations, as predicted by Bever and Wang [9,34]. Therefore, it would be extremely interesting to study nuclear exchange by anastomosis as an essential mechanism to maintain genetic variation in *G. etunicatum*.

In the broader context of the heterokaryotic state of *G. etunicatum*, it seems that the functioning of *PLS* within *G. etunicatum* is not hindered by the presence of multiple alleles. The sequence conservation on the amino acid level shows that the majority of allelic diversity is translated into the same amino acid sequence. However, this may not be the case for all loci that show genetic differentiation between nuclei. In the aforementioned case of diminished anastomosis rates between subcultures from the same isolate, genetic segregation was shown to have a significant influence on a phenotypic trait, in this case anastomosis rate [54]. Additional evidence of the life history consequences of genetically differentiated nuclei in AMF is the observation that spores of *G. irregulare* that contain less than a critical number of nuclei are unable to germinate [8]. Regardless of how the interactions of differentiated genes and genomes within the AMF cytoplasm may play out, the major finding of this study is that, at least for the marker *PLS*, a spore might not contain all the genetic variation that can be found in the original hyphal system. 

## Materials and Methods

### Establishment of single spore pot cultures

Spores of *Glomus etunicatum* (synonym *Claroideoglomus etunicatum*) isolate NPI (Native Plant Industries, Salt Lake City) were isolated from calcined clay through wet sieving and rinsed in sterile water. Spores that appeared opaque and dark brown in colour were considered healthy and chosen preferentially for use in the experiment. 

One hundred and fifty 115 ml cone-tainers (Stuewe & Sons, Inc., Corvallis, OR, USA) were filled with a sterilized mixture of 1:1:1 (v/v) perlite, sand, and sandy loam soil collected from the Montreal Botanical Garden. In each cone-tainer, a 1000 µl micropipette tip with the smaller end trimmed off was pushed into the substrate such that the larger end was level with the surface. A germinated tomato seedling (*Solanum lycopersicum* L. cv. Micro-Tom [57] with a single *G. etunicatum* spore placed upon the root tip was then inserted into each micropipette tip, helping to keep the spore in close proximity to the root, and topped with more sterile soil mixture. Cone-tainers were then maintained in a growth chamber (Table S3). 

Following a six-month growth period, 25 plants were chosen at random for spore collection. The roots of these plants were cut into small pieces and wet sieved along with the soil core to isolate spores. Spores were separated from debris of similar size by centrifuging at 1620 g for 2 min in a discontinuous density gradient with a 60% (w/v) sucrose fraction. The spores were collected from the gradient interface with a Pasteur pipette and thoroughly washed with distilled water before being stored in distilled water at 4°C.

### Gene amplification and pyrosequencing of individual spores

Two to three spores from each of three replicate host plants were isolated for further analysis. These spores were individually picked and placed in 1 µl sterile water in a 0.2 ml tube, then crushed using fine forceps, which were flame-sterilized between samples. The entire DNA content of each of the spores was amplified using the GenomiPhi Whole-Genome Amplification Kit (GE Healthcare, Canada) according to manufacturer instructions. PLS sequences were then amplified using Dream*Taq* DNA polymerase (Fermentas) using primers Pol4-A (GAATCCTTCCCAAATTGATCAGAATACTTGTT) and Pol7-B (TAATAATAAAAGCCTTTCAAAAAATCCATCAATA) [33], with added pyrosequencing adaptor and key sequences at the 5’ ends (forward: CCATCTCATCCCTGCGTGTCTCCGACTCAG, reverse: CCTATCCCCTGTGTGCCTTGGCAGTCTCAG). The reaction was performed in 50 µl volumes containing 0.2 mM dNTPs, 0.5 µmol of each primer and the PCR buffer. PCR was carried out for 34 cycles (95°C for 30 sec, 50°C for 30 sec, 72°C for 1 min; preceded by an initial 2 min denaturation at 94°C and followed by a 10 min hold at 72°C) on a Mastercycler ep gradient S thermal cycler (Eppendorf). The PCR product was checked on an electrophoresis gel to ensure successful amplification of the gene, then purified using a MinElute PCR Purification Kit (Qiagen) according to manufacturer instructions. These purified samples were then sent to the Genome Quebec Innovation Centre (McGill University, Montreal) for pyrosequencing using the GS FLX Titanium emPCR kit (Roche 454 Life Science) with lib-L chemistry (1/8 run per sample).

### Pyrosequencing of parent spores

Approximately 1000 spores of the parental culture of the AMF *G. etunicatum* isolate NPI were collected as described above in the section on the establishment of single spore pot cultures. These spores were subjected to DNA extraction using DNeasy Plant Mini Kit (Qiagen, Canada) according to the manufacture’s recommendations. *PLS* sequences were then amplified using HotStarTaq DNA-Polymerase*‎* (Qiagen, Canada) using the primers Pol4-A and Pol7-B as described above, but with the addition of a Multiplex Identifier (MID) sequence: ACGAGTGCGT on Pol4-A. The reaction was performed in 50 µl volumes containing 0.2 mM dNTPs, 0.5 µmol of each primer and the PCR buffer. PCR purification and 454 sequencing was performed as described above except that the sample was pooled with 19 samples and a 1/8 run was performed, yielding 2422 raw reads. 

### Pyrosequencing analysis

All analyses were performed in Mothur v.1.22 [41], unless specified otherwise. Stringent checks were performed on the raw pyrosequencing data to remove low-quality reads and minimize sequencing errors that could have been introduced during the pyrosequencing process [42,43]. Eliminated reads included those that (i) did not perfectly match the adaptor and primer sequences, (ii) had ambiguous bases, (iii) had a quality score below an average of 35 in a window of 50 bp, (iv) contained homopolymer lengths greater than 8 bp. Reads that passed quality control were preclustered following Huse et al. [48]. Chimeric molecules can form *in vitro* during PCR [58] or pyrosequencing [50]. To control for this artefactual genetic variation, we removed chimeric reads that did not match a database of previously obtained, Sanger sequenced *PLS* sequences [22] with at least 90% bootstrap support using the program Chimeraslayer [50], as implemented in Mothur v.122. 

The remaining clusters are henceforth referred to as ‘alleles’. We performed rarefaction analyses for the entire dataset and for the ‘plant’ and ‘spore’ subsets. A clear asymptote in the rarefaction analysis is an indication that sampling was representative of the actual diversity at the PLS locus. We calculated diversity indices for plants and spores, including the total number of alleles and the Chao1 diversity index (which is a measure of the minimum richness). To explore diversity between spores, we calculated the Jaccard similarity coefficient (jclass) based on the observed richness and the Yue & Clayton (thetaYC) measure of dissimilarity between the structures of two communities. The statistical significance of these latter two coefficients was calculated using the (un)weighted Unifrac distance metric [59]. Statically significant differences between spore and plant communities (and community structures) were determined using the nonparametric analysis of molecular variance (AMOVA) [60-62], and a distance-based version of Bartlett's test for homogeneity of variance (HOMOVA) [63]. 

### Cloning and Sanger sequencing of *PLS* alleles

Spores of *G. etunicatum* isolate NPI grown in leek pot cultures in a greenhouse were removed from soil via wet-sieving as described above. Genomic DNA was obtained *en masse* from approximately 150 spores using a DNeasy Plant Mini Kit (Qiagen) according to the manufacturer's directions. The DNA was then used as a template to amplify PLS using high fidelity *Pfu* DNA polymerase (Fermentas) and specific primers FwdPOL4 and RevPOL7 (see above). The reaction was performed in a 50 μl volume containing 1.25 units *Pfu*, 0.2 mM dNTPs, 0.5 μM of each primer and the PCR buffer. PCR was carried out for 34 cycles (94°C for 30 sec, 54°C for 30 sec, 72°C for 1 min; preceded by an initial 2 min denaturation at 94°C and followed by a 10 min hold at 72°C) on a Mastercycler ep gradient S thermal cycler (Eppendorf). After the PCR product was checked on an electrophoresis gel to ensure successful amplification, the amplified gene was cloned using a CloneJET PCR Cloning Kit (Fermentas), according to the manufacturer’s instructions. Three hundred and six bacterial colonies containing the PLS insert were subcultured on Luria-Bertani (LB) medium [64] containing 100 mg/l ampicillin, and sequenced at the Genome Quebec Innovation Centre (McGill University, Montreal) using pJET1.2 forward and reverse sequencing primers (Fermentas).

### Sanger sequencing analysis

 Sequence chromatograms were visualized using the program Finch TV (version 1.4.0, Geospiza Inc.); those which were low-quality, incomplete, or which contained plasmid sequences were removed from the analysis. Sequences were aligned using MEGA4 [65], corrected by hand, and deposited in Genbank with accession numbers GQ325050-GQ325231. In determining the number of distinct variants found, two estimates were made; a conventional estimate, in which all single nucleotide polymorphisms (SNPs) were assumed to have truly occurred in the sequences, and a conservative estimate in which all SNPs occurring in only one sequence were dismissed as artefactual. The online program DNAcollapser [66] was used to collapse the sequences to distinct (non-identical) variants. In order to predict the maximum number of distinct variants in the NPI strain, a Monte Carlo simulation was carried out. One thousand replicates of the Monte Carlo simulation were carried out for each estimate using the R platform for statistical computing (www.r-project.org/). For each graph, 95% confidence intervals (mean value ± 1.96 × standard deviation) of the simulated values were also plotted.

### Data depositions

Sanger sequenced nucleotide sequences have been deposited in GenBank under the accession numbers GQ325050-GQ325231 and raw pyrosequencing reads are available at the Sequence Read Archive under Bioproject number PRJNA185186

## Supporting Information

Figure S1 **Rarefaction analyses for all spores pooled together (all), spores pooled by plant (A-B-C), and each individual spore (A1-A2-A4-B1-B2-B4-C1-C2) and the parent isolate. The number of recovered alleles (y axis, blue line, 95 % confidence intervals indicated by vertical lines) is compared to the maximum Chao1 value [44], which is the estimated minimum richness for each group (solid black line, 95 % confidence intervals in dotted lines).**
(DOCX)

Figure S2 **Nucleotide diversity π along the first intron and second exon of the *PLS* marker.**
(DOCX)

Figure S3 **Allele distributions of PLS alleles recovered from pyrosequencing runs on spores and the parent isolate, for a) all alleles that occur three times or more in the dataset and b) the four most abundant alleles.**
(DOCX)

Figure S4 **Rarefaction analysis of pooled alleles that were found in pyrosequencing runs from parent and offspring spores, as a function of sampling depth in sequences. Orange shading indicates 95% confidence intervals.**
(DOCX)

Table S1 **Absolute (a) and relative (r) numbers of reads excluded in quality control (QC).**
(DOCX)

Table S2 **Summary of results from analyses of *PLS* Sanger sequenced data.**
(DOCX)

Table S3 **Environmental conditions under which tomato seedlings were grown.**
(DOC)
