# Supplementary material for: Allelic Differences within and among Sister Spores of the Arbuscular Mycorrhizal Fungus Glomus etunicatum Suggest Segregation at Sporulation
Source: PLoS One. 2013 Dec 26;8(12):e83301. doi: 10.1371/journal.pone.0083301 (PMC3873462; doi:10.1371/journal.pone.0083301)
Supplement: Figure S1 — Rarefaction analyses for all spores pooled together (all), spores pooled by plant (A-B-C), and each individual spore (A1-A2-A4-B1-B2-B4-C1-C2) and the parent isolate. The number of recovered alleles (y axis, blue line, 95 % confidence intervals indicated by vertical lines) is compared to the maximum Chao1 value [44], which is the estimated minimum richness for each group (solid black line, 95 % confidence intervals in dotted lines). (DOCX) [file pone.0083301.s001.docx]

**Supplementary Figure 1**

Rarefaction analyses


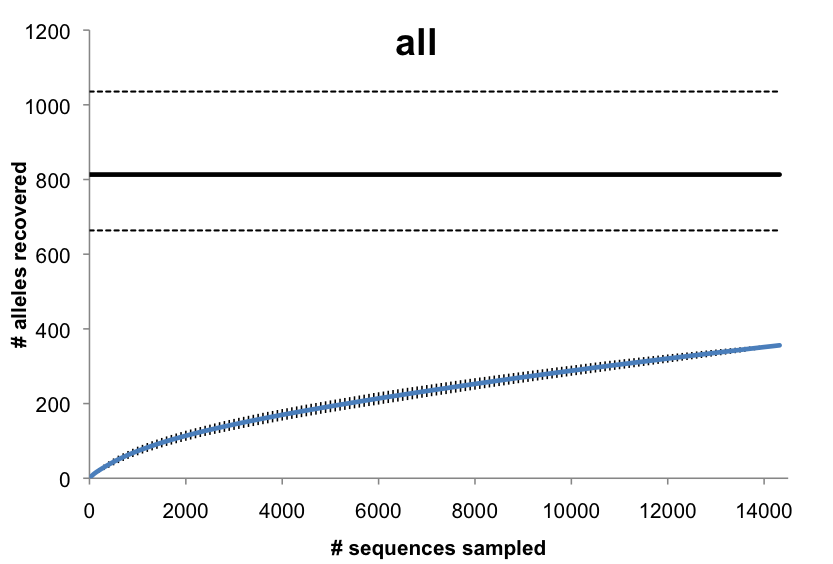


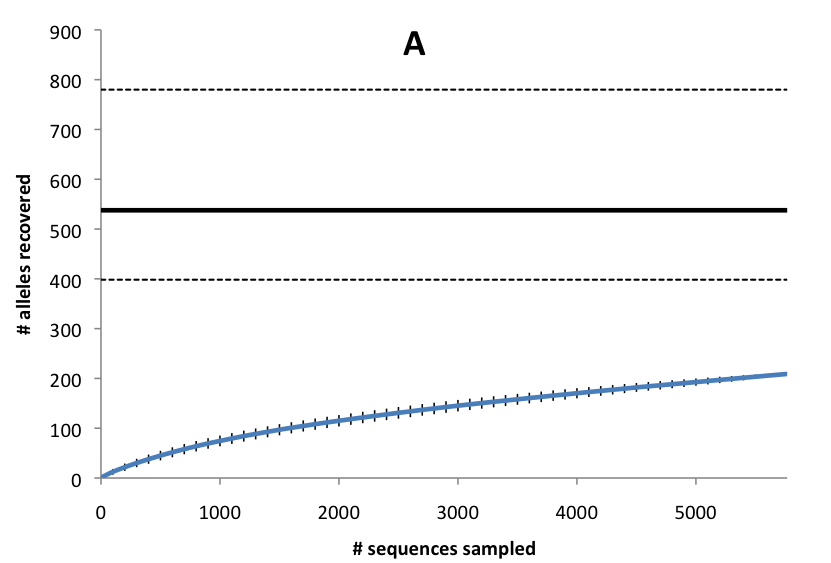


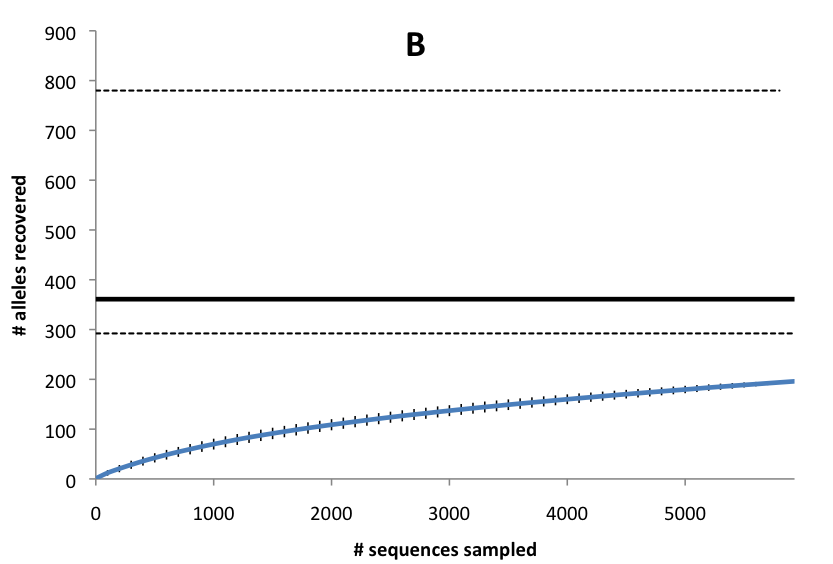


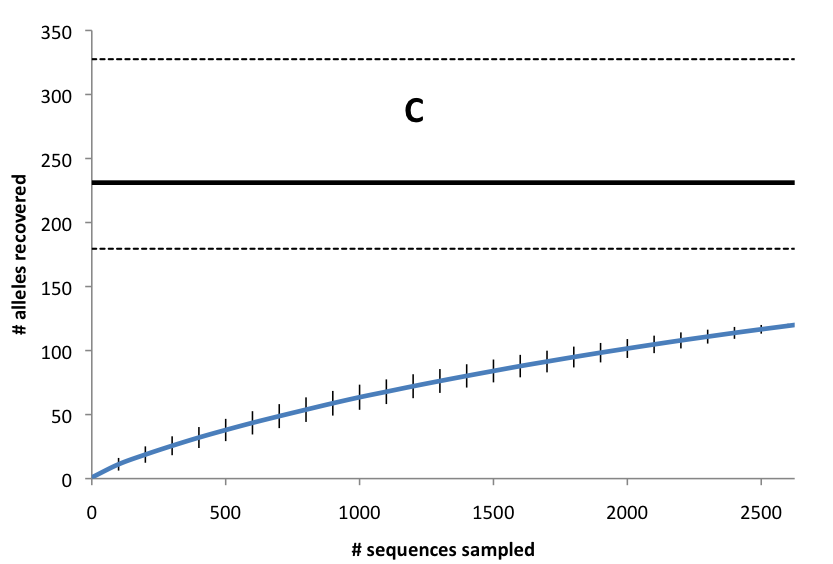


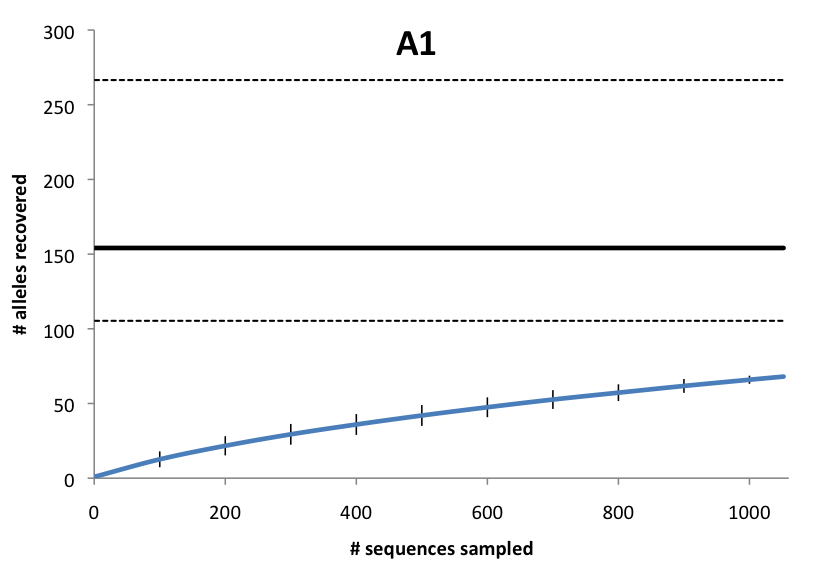


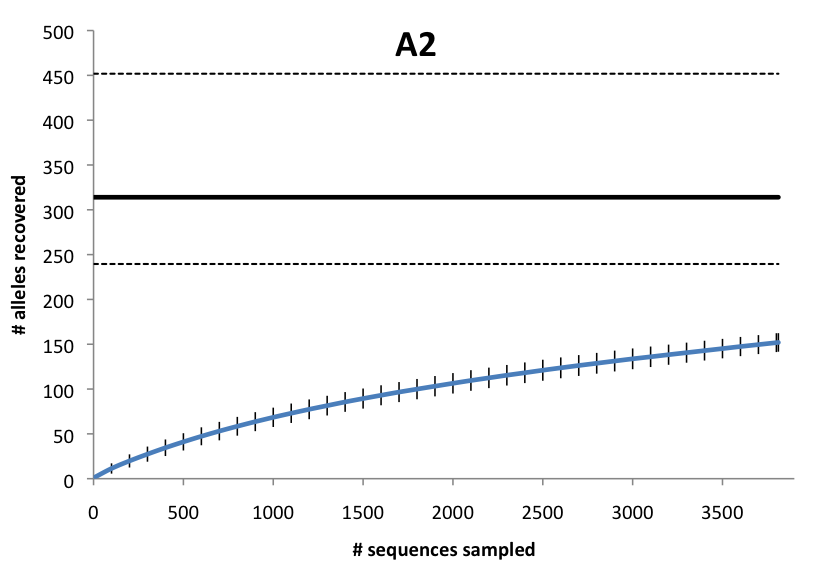


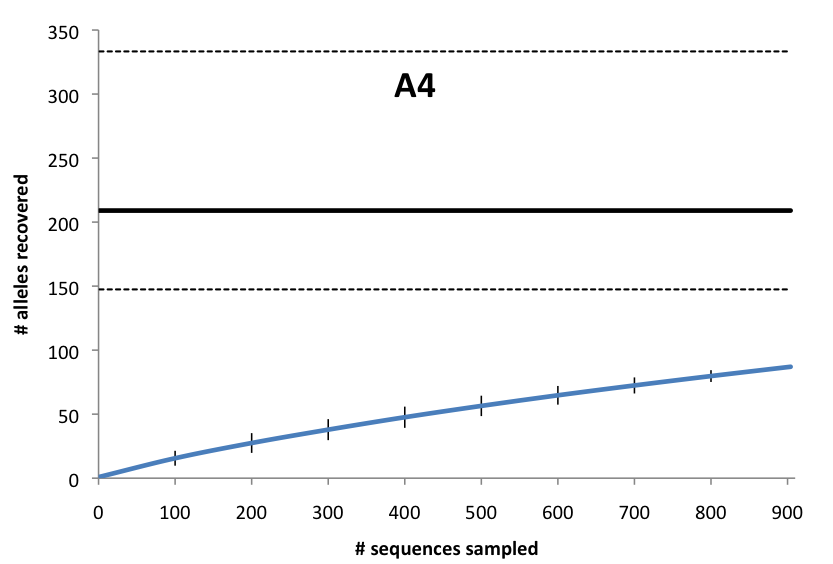


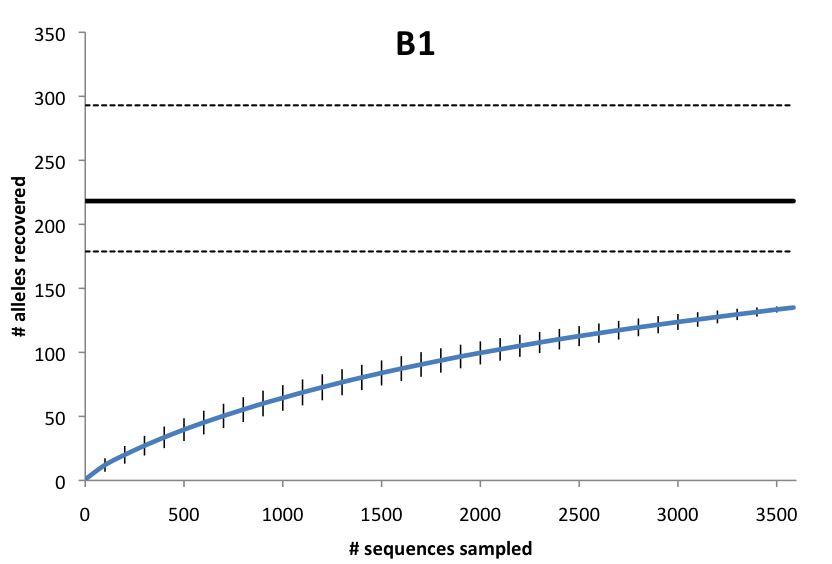


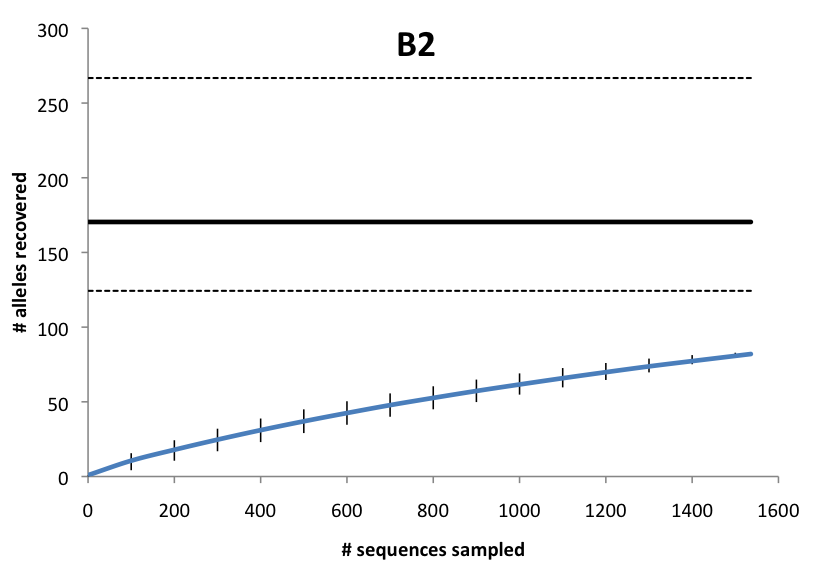


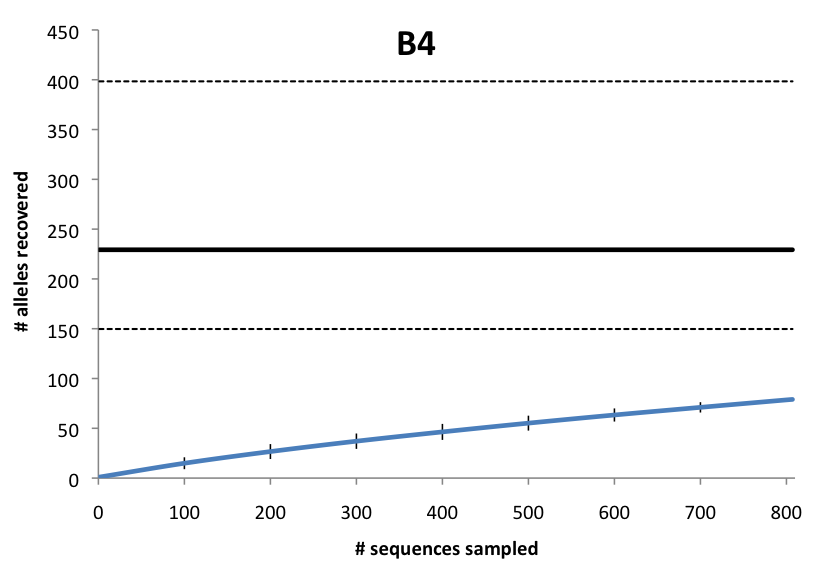


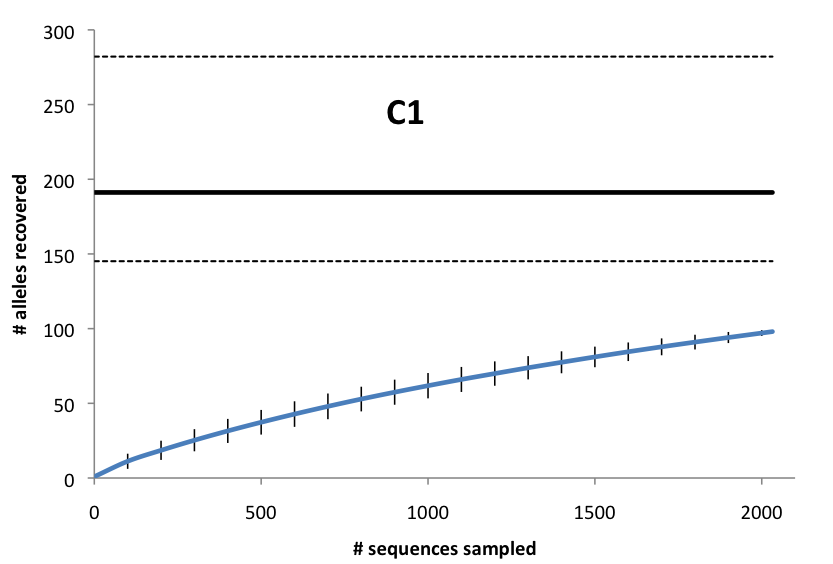


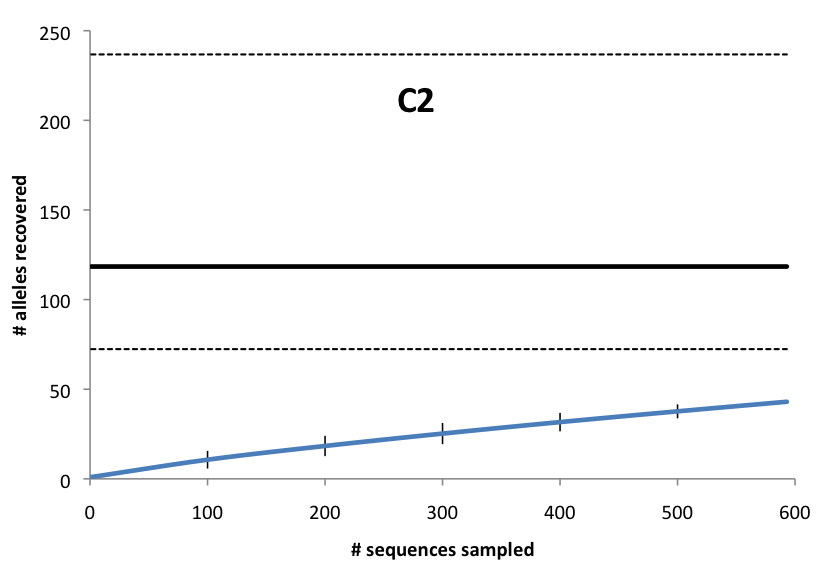


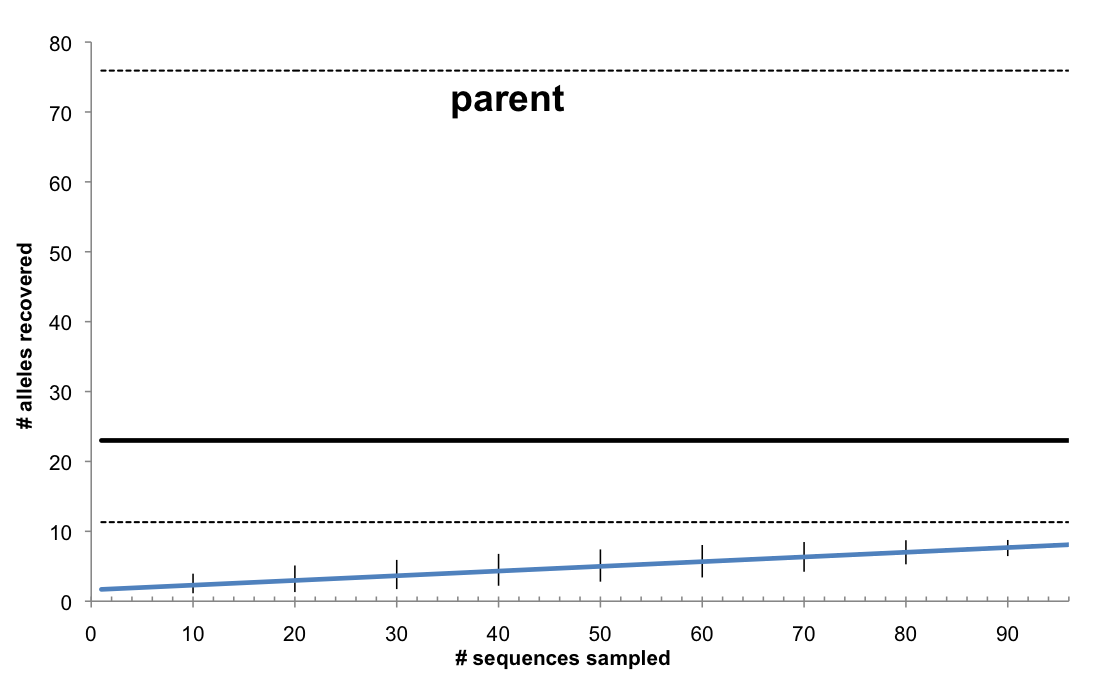


Figure S1 Rarefaction analyses for all spores pooled together (all), spores pooled by plant (A-B-C), and each individual spore (A1-A2-A4-B1-B2-B4-C1-C2) and the parent isolate. The number of recovered alleles (y axis, blue line, 95 % confidence intervals indicated by vertical lines) is compared to the maximum Chao1 value ([Chao *et al.* 2005](#_ENREF_30)), which is the estimated minimum richness for each group (solid black line, 95 % confidence intervals in dotted lines).
