# Supplementary material for: Allelic Differences within and among Sister Spores of the Arbuscular Mycorrhizal Fungus Glomus etunicatum Suggest Segregation at Sporulation
Source: PLoS One. 2013 Dec 26;8(12):e83301. doi: 10.1371/journal.pone.0083301 (PMC3873462; doi:10.1371/journal.pone.0083301)
Supplement: Figure S2 — Nucleotide diversity π along the first intron and second exon of the PLS marker. (DOCX) [file pone.0083301.s002.docx]

**Supplementary Figure 2**


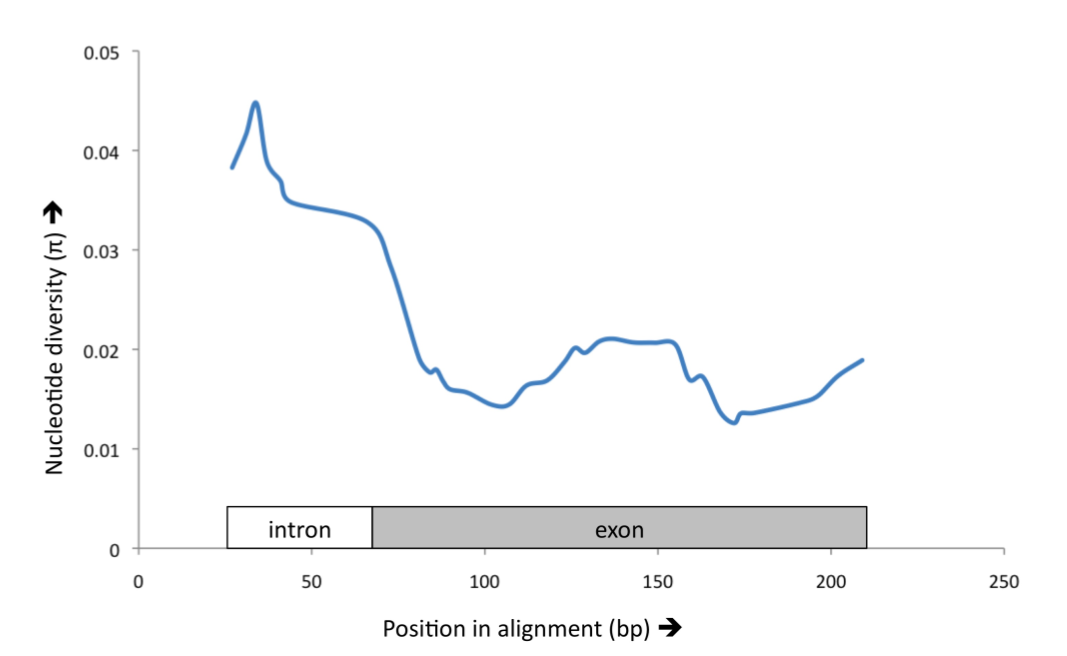


Figure S2 Nucleotide diversity π along the first intron and second exon of the *PLS* marker.
