# Supplementary material for: Allelic Differences within and among Sister Spores of the Arbuscular Mycorrhizal Fungus Glomus etunicatum Suggest Segregation at Sporulation
Source: PLoS One. 2013 Dec 26;8(12):e83301. doi: 10.1371/journal.pone.0083301 (PMC3873462; doi:10.1371/journal.pone.0083301)
Supplement: Figure S3 — Allele distributions of PLS alleles recovered from pyrosequencing runs on spores and the parent isolate, for a) all alleles that occur three times or more in the dataset and b) the four most abundant alleles. (DOCX) [file pone.0083301.s003.docx]

**Supplementary Figure 3**


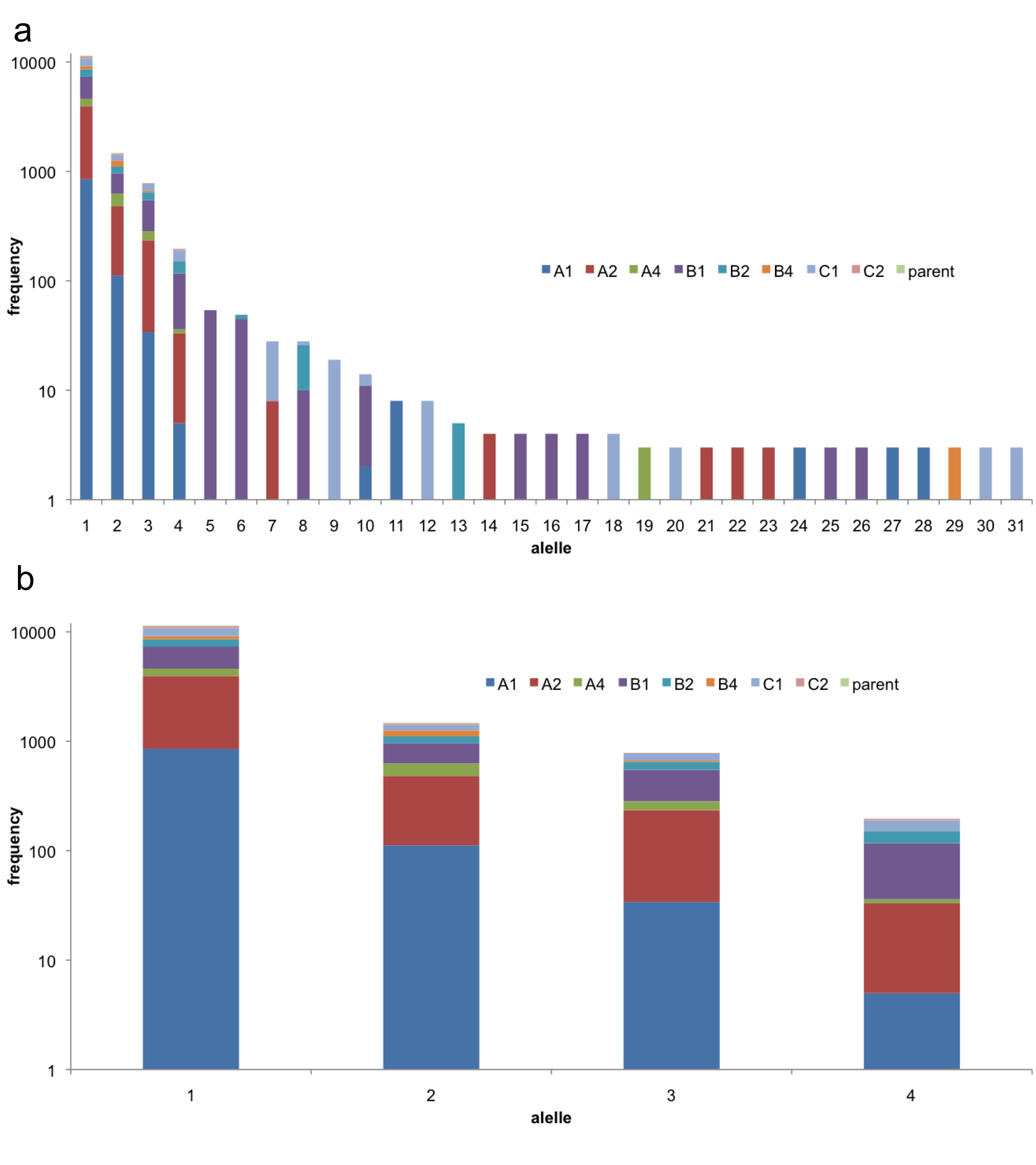


Figure S3 Allele distributions of PLS alleles recovered from pyrosequencing runs on spores and the parent isolate, for a) all alleles that occur three times or more in the dataset and b) the four most abundant alleles.
