# Supplementary material for: Allelic Differences within and among Sister Spores of the Arbuscular Mycorrhizal Fungus Glomus etunicatum Suggest Segregation at Sporulation
Source: PLoS One. 2013 Dec 26;8(12):e83301. doi: 10.1371/journal.pone.0083301 (PMC3873462; doi:10.1371/journal.pone.0083301)
Supplement: Figure S4 — Rarefaction analysis of pooled alleles that were found in pyrosequencing runs from parent and offspring spores, as a function of sampling depth in sequences. Orange shading indicates 95% confidence intervals. (DOCX) [file pone.0083301.s004.docx]

**Supplementary Figure 4**

Figure S4 rarefaction analysis of pooled alleles that were found in pyrosequencing runs from parent and offspring spores, as a function of sampling depth in sequences. Orange shading indicates 95% confidence intervals.
