# Supplementary material for: Allelic Differences within and among Sister Spores of the Arbuscular Mycorrhizal Fungus Glomus etunicatum Suggest Segregation at Sporulation
Source: PLoS One. 2013 Dec 26;8(12):e83301. doi: 10.1371/journal.pone.0083301 (PMC3873462; doi:10.1371/journal.pone.0083301)
Supplement: Table S1 — Absolute (a) and relative (r) numbers of reads excluded in quality control (QC). (DOCX) [file pone.0083301.s005.docx]

**Supplementary Table 1**

Absolute (a) and relative (r) numbers of reads excluded in quality control (QC).

| Spore | Raw | | Trim, aligned | | Screen, unique | | Manual  alignment | | Remove  Chimeras | | Filter,  unique | | Precluster | | Final | |
| --- | --- | --- | --- | --- | --- | --- | --- | --- | --- | --- | --- | --- | --- | --- | --- | --- |
|  | a^1^ | r^2^ | a^1^ | r^2^ | a^1^ | r^2^ | a^1^ | r^2^ | a^1^ | r^2^ | a^1^ | r^2^ | a^1^ | r^2^ | a^1^ | r^2^ |
|  |  |  |  |  |  |  |  |  |  |  |  |  |  |  |  |  |
| A1 | 11242 | 1.00 | 11102 | 0.99 | 3719 | 0.33 | 988 | 0.09 | 913 | 0.08 | 910 | 0.08 | 263 | 0.02 | 148 | 0.01 |
| A2 | 43001 | 1.00 | 42565 | 0.99 | 18302 | 0.43 | 5975 | 0.14 | 2987 | 0.07 | 2980 | 0.07 | 790 | 0.02 | 445 | 0.01 |
| A4 | 11031 | 1.00 | 10948 | 0.99 | 5192 | 0.47 | 1156 | 0.10 | 828 | 0.08 | 828 | 0.08 | 311 | 0.03 | 161 | 0.01 |
| B1 | 22711 | 1.00 | 22578 | 0.99 | 10345 | 0.46 | 3141 | 0.14 | 3125 | 0.14 | 3092 | 0.14 | 756 | 0.03 | 417 | 0.02 |
| B2 | 5051 | 1.00 | 5023 | 0.99 | 3240 | **0.64**^3^ | 1691 | **0.33**^3^ | 1394 | **0.28**^3^ | 1379 | **0.27**^3^ | 343 | **0.07**^3^ | 174 | 0.03 |
| B4 | 21942 | 1.00 | 20887 | 0.95 | 6868 | 0.31 | 1456 | 0.07 | 677 | **0.03**^3^ | 676 | **0.03**^3^ | 279 | 0.01 | 144 | 0.01 |
| C1 | 11920 | 1.00 | 11815 | 0.99 | 5911 | 0.50 | 1866 | 0.16 | 1860 | 0.16 | 1816 | 0.15 | 475 | 0.04 | 259 | 0.02 |
| C2 | 4899 | 1.00 | 4859 | 0.99 | 1967 | 0.40 | 575 | 0.12 | 518 | 0.11 | 518 | 0.11 | 159 | 0.03 | 83 | 0.02 |
|  |  |  |  |  |  |  |  |  |  |  |  |  |  |  |  |  |
| Average | 16475 | 1.00 | 16222 | 0.99 | 6943 | 0.44 | 2106 | 0.14 | 1538 | 0.12 | 1525 | 0.12 | 422 | 0.03 | 229 | 0.02 |
| St.Dev | 12619 | 1.00 | 12445 | 0.01 | 5259 | 0.10 | 1740 | 0.08 | 1029 | 0.08 | 1020 | 0.07 | 234 | 0.02 | 134 | 0.01 |
|  |  |  |  |  |  |  |  |  |  |  |  |  |  |  |  |  |
| ^1^ absolute numbers of reads | | | | | | | | | | | | | | | | |
| ^2^ relative number of reads expressed as the proportion of initial (‘raw’) number of reads | | | | | | | | | | | | | | | | |
| ^3^ cases in which the relative number of reads^2^ is smaller or larger than the average ± standard deviation for that QC step | | | | | | | | | | | | | | | | |
