# Supplementary material for: Allelic Differences within and among Sister Spores of the Arbuscular Mycorrhizal Fungus Glomus etunicatum Suggest Segregation at Sporulation
Source: PLoS One. 2013 Dec 26;8(12):e83301. doi: 10.1371/journal.pone.0083301 (PMC3873462; doi:10.1371/journal.pone.0083301)
Supplement: Table S2 — Summary of results from analyses of PLS Sanger sequenced data. (DOCX) [file pone.0083301.s006.docx]

**Supplementary Table 2**

Summary of results from analyses of *PLS* Sanger sequenced data.

|  | Conventional^1^ | Conservative^1^ |
| --- | --- | --- |
|  |  |  |
| Total # sequences | 182 | 182 |
| Sequence length (bp) | 610 | 602 |
| Alleles | 113 | 103 |
| Variable sites | 66 | 38 |
| Allele distribution |  |  |
| allele frequency |  |  |
| 1 | 79 | 66 |
| 2 | 17 | 18 |
| 3 | 9 | 10 |
| 4 | 4 | 2 |
| 5 | 1 | 3 |
| 6 | 0 | 1 |
| 7 | 3 | 3 |

^1^ conventional: every new SNP leads to a different allele, conservative: alleles are only counted if SNPs occurs more than once
