# Supplementary material for: Allelic Differences within and among Sister Spores of the Arbuscular Mycorrhizal Fungus Glomus etunicatum Suggest Segregation at Sporulation
Source: PLoS One. 2013 Dec 26;8(12):e83301. doi: 10.1371/journal.pone.0083301 (PMC3873462; doi:10.1371/journal.pone.0083301)
Supplement: Table S3 — Environmental conditions under which tomato seedlings were grown. (DOC) [file pone.0083301.s007.doc]

**Supplementary Table 3**

Environmental conditions under which tomato seedlings were grown.

| Time of Day | Temp. (°C) | Humidity (%) | Fluorescent bulbs BBulbBBulbs | Incandescent bulbs |
| --- | --- | --- | --- | --- |
| 5h30 | 20 | 60 | 0 | 2 |
| 6h00 | 23 | 70 | 2 | 2 |
| 7h00 | 23 | 70 | 4 | 4 |
| 19h00 | 20 | 60 | 2 | 2 |
| 21h30 | 18 | 55 | 0 | 2 |
| 22h00 | 18 | 55 | 0 | 0 |
